# Resistive Switching of Plasma–Treated Zinc Oxide Nanowires for Resistive Random Access Memory

**DOI:** 10.3390/nano6010016

**Published:** 2016-01-13

**Authors:** Yunfeng Lai, Wenbiao Qiu, Zecun Zeng, Shuying Cheng, Jinling Yu, Qiao Zheng

**Affiliations:** 1 School of Physics and Information Engineering, Fuzhou University, Fuzhou 350108, China; albert_29@163.com (W.Q.); zecunzeng@163.com (Z.Z.); sycheng@fzu.edu.cn (S.C.); jlyu@semi.ac.cn (J.Y.); 2004_zhengqiao@163.com (Q.Z.); 2 Jiangsu Collaborative Innovation Center of Photovoltaic Science and Engineering, Changzhou University, Changzhou 213164, China

**Keywords:** resistive switching, plasma treatment, ZnO nanowires, self-rectification

## Abstract

ZnO nanowires (NWs) were grown on Si(100) substrates at 975 °C by a vapor-liquid-solid method with ~2 nm and ~4 nm gold thin films as catalysts, followed by an argon plasma treatment for the as-grown ZnO NWs. A single ZnO NW–based memory cell with a Ti/ZnO/Ti structure was then fabricated to investigate the effects of plasma treatment on the resistive switching. The plasma treatment improves the homogeneity and reproducibility of the resistive switching of the ZnO NWs, and it also reduces the switching (set and reset) voltages with less fluctuations, which would be associated with the increased density of oxygen vacancies to facilitate the resistive switching as well as to average out the stochastic movement of individual oxygen vacancies. Additionally, a single ZnO NW–based memory cell with self-rectification could also be obtained, if the inhomogeneous plasma treatment is applied to the two Ti/ZnO contacts. The plasma-induced oxygen vacancy disabling the rectification capability at one of the Ti/ZnO contacts is believed to be responsible for the self-rectification in the memory cell.

## 1. Introduction

Resistive random access memory (RRAM) is capable of reversible switching between high resistance state (HRS) and low resistance state (LRS) under suitable electrical stress, which has been confirmed to be closely associated with defects in the memory [1,2,3]. The significance of controlling defects in the memory cells would be more pronounced in the nano-era, since the memory cells shrink their physical dimension for high density integration [4,5,6]. As for the storage medium of the RRAMs, zinc oxide (ZnO), a wide band gap semiconductor with abundant intrinsic defects, has recently attracted considerable attention for its potential applications in transparent RRAMs due to its excellent resistive switching [7,8,9]. Understanding and controlling the defects in the ZnO nanowire (NW)–based RRAM are thus worthwhile [6,10,11,12].

In order to further improve storage performance such as the retention, stability and homogeneity of switching properties, several schemes have been attempted to control defects in the storage medium and on the interface [13,14,15,16,17,18,19]. Doping certain elements or embedding nano-particles into the storage medium seems to be a feasible way to rearrange the defects and the storage performance could thus be improved [13,14,15]. An alternative way is to change interfacial defects by selecting suitable electrodes or by stacking a different storage medium to form a hetero-interface [16,17,18,19]. Plasma treatment is a widely accepted technique for surface modification due to its physical effects as well as chemical ones on the treated materials. It also exhibits low cost, high efficiency, high control accuracy and good treatment homogeneity to normally modify the defects. The resistive switching of RRAMs might be adjusted as well. However, the effects of plasma treatment on the switching properties of RRAMs are scarcely investigated [6,20]. We therefore fabricated single ZnO NW–based RRAMs with a Ti/ZnO NW/Ti structure. Argon plasma treatment was also applied to the ZnO NW to evaluate the effects of plasma treatment on the switching properties of the RRAMs.

## 2. Results and Discussion

The switching properties are critically important for the practical applications of the RRAMs. We hereby evaluate the effects of plasma treatment on the switching properties of the ZnO NW–based memory according to the homogeneity and reproducibility of the switching parameters, the data retention and the embedded self-rectification capability.

### 2.1. Enhanced Homogeneity and Reproducibility

**Figure 1:**
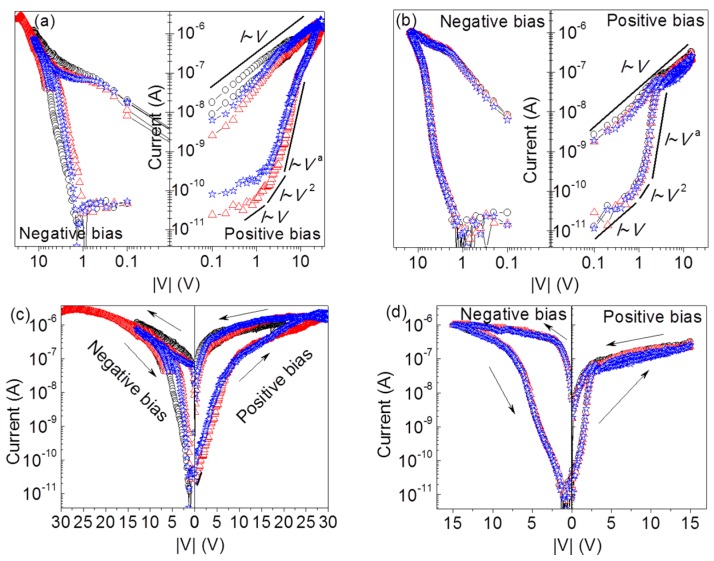
Reproducible voltage-biased current-voltage (*I*–*V*) curves of the ZnO nanowires (ZnO NW)–based memories (**a**,**c**) without and (**b**,**d**) with argon plasma treatment. Blue (☆), black (○) and red (△) symbols respectively represent the 8th, 18th and 28th switching cycles.

**Figure 2:**
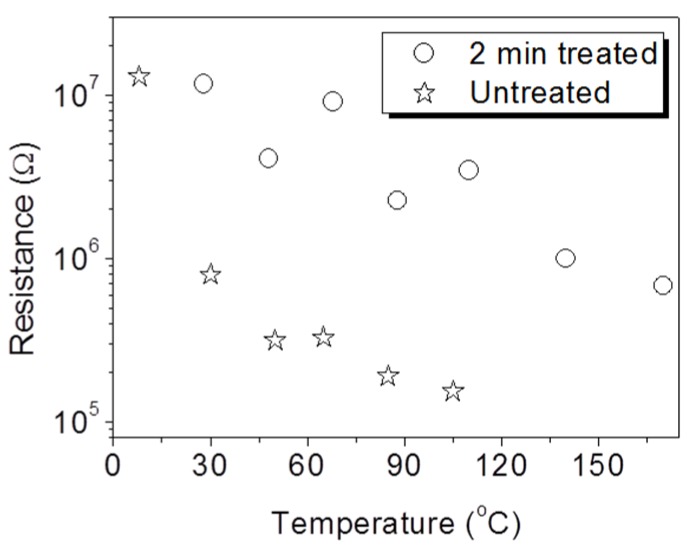
Temperature-dependent resistance of the low resistance state (LRS) memories with and without plasma treatment.

Storage properties of a RRAM are criterions to estimate the effectiveness of plasma treatment. Figure 1 shows the voltage-biased current-voltage (*I*–*V*) curves of the plasma-treated and untreated memory cells at every 10 switching cycles (marked with blue, black and red symbols). To improve the readability of the *I*–*V* curves, the curves from both the plasma-treated samples and the untreated ones are shown on log-log scale (Figure 1a,b) and are respectively accompanied with curves on normally log-linear scale (Figure 1c,d). Both memory cells exhibit bipolar resistive switching with decreased set and reset voltages by plasma treatment. In addition, the repeatability of switching behavior is significantly enhanced by plasma treatment. To get insights into the switching mechanism, the *I*–*V* curves on log-log scale were studied. Each *I*–*V* curve during the set period is composed of three portions with different slopes (the Ohmic region with *I*~*V*, the Child’s square-law region with *I*~*V*^2^, and the exponentially increased current region with *I*~*V*^a^), showing a typical feature of space-charge-limited conduction (SCLC) [21,22]. The oxygen vacancy-assisted filaments and metal-dominated filaments could be closely associated with the electrical switching between the HRS and the LRS [12,23,24]. Figure 2 shows the temperature-dependent resistance of the LRS memory cells. The decreasing resistance upon the increased temperature is in good agreement with a semiconductor conduction but denies a metallic conduction [6]. We thus deduce that the rupture and formation of oxygen vacancy-assisted filaments should be respectively responsible for the reset and the set process. The formation of TiO*_x_* at the Ti/ZnO interface is inevitable in this work due to a much higher enthalpy of the formation for TiO_2_ (−944 kJ/mol) than that for ZnO (−350 kJ/mol) [10]. During the revisable resistance switching, the oxygen atoms in the TiO*_x_* would also migrate back and forth between the TiO*_x_* and the ZnO upon the applied electric fields to facilitate the rupture and formation of the conductive filaments.

**Figure 3:**
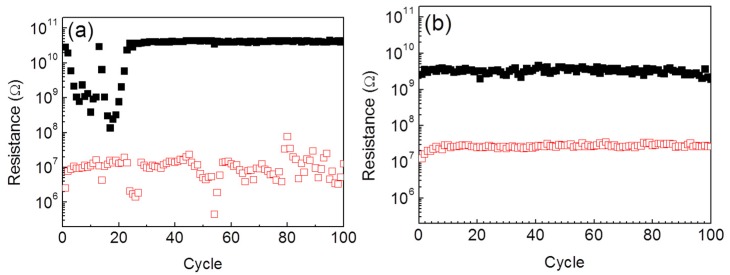
Endurance tests of the ZnO NW–based memories (**a**) without and (**b**) with argon plasma treatment.

The reversible switching tests were subsequently carried out for another 100 cycles to estimate the endurance of the memory cells. Figure 3a shows the endurance of the memory cell without plasma treatment, and the plasma-treated one is shown in Figure 3b. At the first 30 switching cycles, the HRS of the untreated memory is unstable with fluctuations in resistance. It gets reproducible during the left 70 cycles. The LRS of the untreated memory starts with acceptably reproducible resistances but its repeatability becomes worse after 80 switching cycles. However, both the HRS and the LRS of the plasma-treated memory are reproducible with ignorable fluctuations in resistance over the 100 switching cycles, though the resistance ratio between the HRS and the LRS is reduced by plasma treatment.

**Figure 4:**
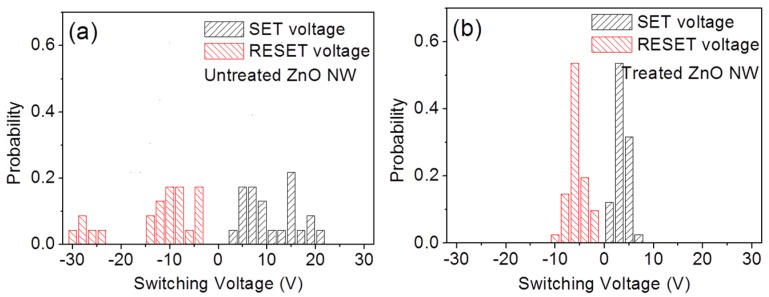
Distributions of switching voltages of the ZnO NW–based memories (**a**) without and (**b**) with argon plasma treatment.

For the practical applications of the RRAMs, the distributions of the set and reset voltages (also known as switching voltages) from different cells should be as narrow as possible to ensure operation reliability. The distributions of the switching voltages were then analyzed and shown in Figure 4. For the untreated ZnO NW, the reset voltages spanning from −3 V to −31 V and the set voltages spanning from 3 to 22 V can be observed without obvious peaking voltages. However, for the plasma-treated ZnO NW memory, both the reset voltages and the set voltages present normal distributions and respectively span from −1.5 to −10 V and from 0.5 to 8 V with peaking at −7 and 3 V, which indicates that the switching voltages decrease by plasma treatment with an increase in homogeneity.

### 2.2. Improved Data Retention

**Figure 5:**
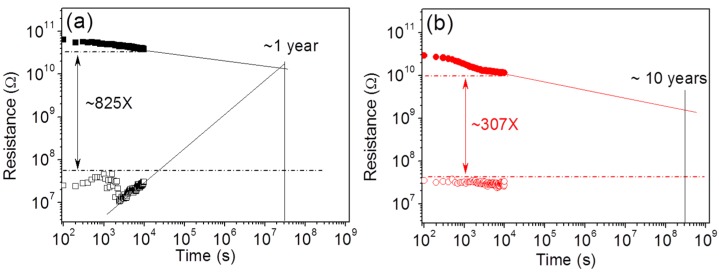
Data retention of the ZnO NW–based memories (**a**) without and (**b**) with argon plasma treatment.

To further clarify the storage performance, the data retention of the memories was evaluated as shown in Figure 5. Plasma treatment reduces the resistance ratio between the two binary storage states but extends the data retention from less than one year to over 10 years by stabilizing the LRS resistance.

### 2.3. Self-Rectification of the RRAM Cells

**Figure 6:**
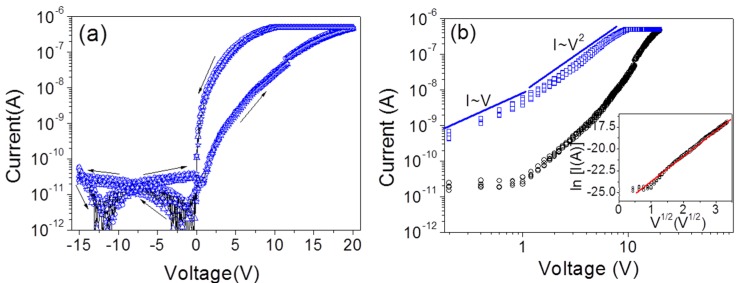
(**a**) Reproducible asymmetric *I*–*V* curves of single ZnO NW and (**b**) the *I*–*V* curves at positive bias on log-log scale with the inset fitting of $\sqrt{V}\sim \ln(I)$ for the high resistance state (HRS).

**Figure 7:**
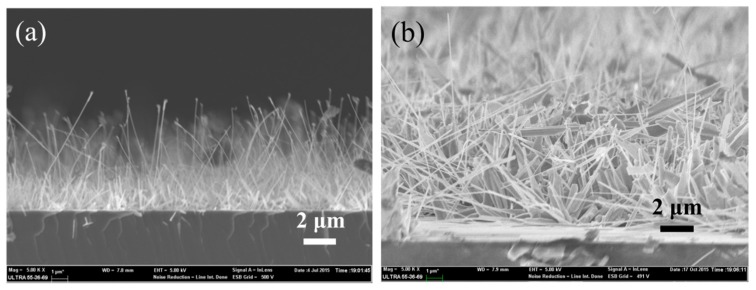
Cross-sectional scanning electron microscope (SEM) images of the ZnO NWs on the silicon substrates with gold thicknesses of (**a**) ~2 nm and (**b**) ~4 nm.

With the demand of ultra-high density integration from the memory industry, crossbar arrays as a feasible architecture for future RRAMs have attracted much attention in recent years [25,26,27]. However, the sneak-path issue is a key challenge for this scheme, since the read-out sense margin could be reduced by the undesired leakage current through the unselected memory cells. To solve this problem, the *I*–*V* curve of the LRS RRAM cell should be non-linear, which could be realized by the integration of a memory resistor and a rectification diode [28]. Except for the almost symmetric *I*–*V* characteristic shown in Figure 1, the asymmetric *I*–*V* characteristic could also be observed for the ZnO nanowires grown with ~4 nm gold thin films as shown in Figure 6a. The current ratio under the positive bias and the negative bias may reach 10,000, indicating an integrated self-rectification in the memory cell. When it comes to the switching mechanism, the *I*–*V* curves were replotted on log-log scale as shown in Figure 6b. An Ohmic region and a Child’s law region form the *I*–*V* curves of the LRS RRAM cell, which is in agreement with the SCLC mechanism [21,22]. However, the SCLC mechanism does not dominate the conduction in the HRS, as a better linear fitting of $\sqrt{V}\sim \ln(I)$ indicates a Schottky emission dominant conduction [29,30]. Therefore, there should be a Schottky barrier at the Ti/ZnO interface. As we know, the rectification is associated with the interface status. Figure 7 shows the scanning electron microscopy (SEM) images of the as-grown ZnO nanowires with different thicknesses of gold catalyst. Compared with the thin (~2 nm) gold catalyzed nanowires in a lower density with a smaller diameter (see Figure 7a), the thick (~4 nm) gold catalyst produces nanowires in a higher density with a greater diameter (see Figure 7b). Additionally, there are some leaf-like species at the root of the thick (~4 nm) gold catalyzed ZnO nanowires to partially protect the nanowire’s root from the plasma treatment (see Figure 7b). Considering the higher density, the protection effects for the root of the thick gold catalyzed nanowires would be further enhanced. The inhomogeneous interfacial treatment on the two terminals of the thick gold catalyzed nanowires would thus be obtained, instead of a much more uniform treatment throughout the whole nanowire with thin gold catalyst. The gold catalyst normally guides the growth of nanowires during the vapor-liquid-solid synthesis process and could hardly have a direct association with the self-rectification. Consequently, the gold catalyst determines the morphology of the as-grown ZnO nanowires. The high density and the leaf-like species contribute to the inhomogeneous interfacial treatment effects and result in self-rectification as shown in Figure 6a.

### 2.4. Defects in the ZnO NWs

**Figure 8:**
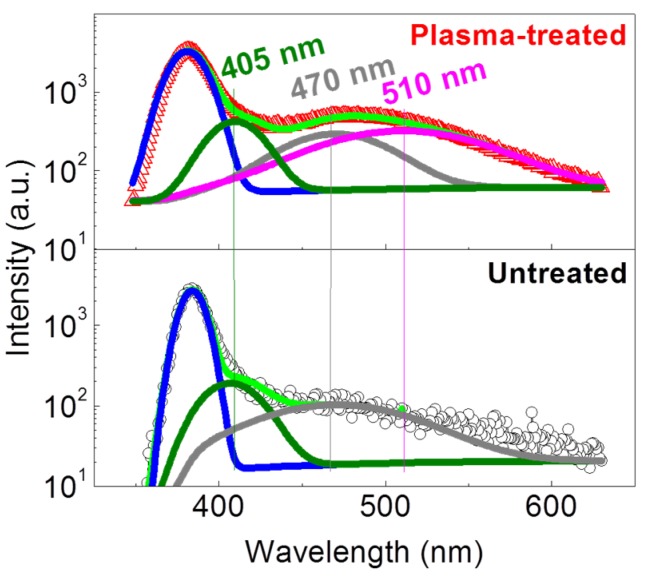
Room-temperature photoluminescence (PL) spectra of the ZnO NWs and their Gaussian components.

Investigation on the defects is helpful for a better understanding of the reversible switching of a RRAM. Figure 8 shows the room-temperature photoluminescence (PL) spectra of the ZnO NWs on log-log scale in order to elucidate weak emissions. Each spectrum is composed of a ~380 nm centered UV emission and a broad visible emission spanning from ~430 nm to ~570 nm. The intensity ratios of the UV emission to the visible emission are about 21.06 and 6.75 for the untreated and the plasma-treated ZnO NWs, respectively, which means the argon plasma treatment introduces defects with visible emissions. We therefore de-convolute them into several symmetrical peaks as shown in Figure 8. For the untreated ZnO NWs, there is a weak ~470 nm centered emission in the visible region, indicating a good crystal structure with few defects. However, except for the ~470 nm centered emission, a ~510 nm centered emission could also be observed for the plasma-treated ZnO NWs, which suggests the increase of deep oxygen vacancy (*V*_o_) by plasma treatment due to the agreement with the electron transition from the deep *V*_o_ level to the top of the valance band [31]. To verify the results, we underwent the experiment three times from three batches of samples. Similar results were observed. During the argon plasma treatment period, the oxygen atoms would be driven out of the ZnO surface by the collision of inert argon ions, which results in an oxygen-deficient surface with oxygen vacancies. The formation of oxygen vacancy-assisted conductive filaments triggers the set process to complete the resistance transition from the HRS to the LRS. If more oxygen vacancies are involved during this period, the uncertainty of the formation of the conductive filaments should be minimized as the distance between the adjacent *V*_o_s is reduced and the electron-hopping between them would be easier. The homogeneity and reproducibility of the resistive switching could thus be improved. Additionally, the increased *V*_o_ density may also effectively average out the stochastic movement of individual V_o_s to improve resistance stability as well as to prolong data retention [32]. Liu Ming also doped HfO_2_ with nitrogen and found that the increased oxygen vacancies play quite similar roles [33]. However, the increased *V*_o_s generally act as dopants in oxide semiconductors to decrease the resistance and result in a slightly suppressed resistance ratio between the HRS and the LRS as what we observe in Figure 3b. As for the self-rectification in the plasma-treated RRAM cell, the effects of plasma treatment on the two back-to-back connected Schottky barriers at the Ti/ZnO contacts should be taken into account. The high *V*_o_ density usually results in the ZnO Fermi level pinning close to the defect level to increase the probability of electron tunneling by narrowing the region of positive space charge [34], and the rectification capability of the Ti/ZnO interface may be disabled. As a result, the plasma treatment weakens the rectification at one Ti/ZnO contact but indeed manifests the rectification capability at another Ti/ZnO contact. The self-rectification phenomenon could thus be observed as shown in Figure 6.

## 3. Experimental Section

**Figure 9:**
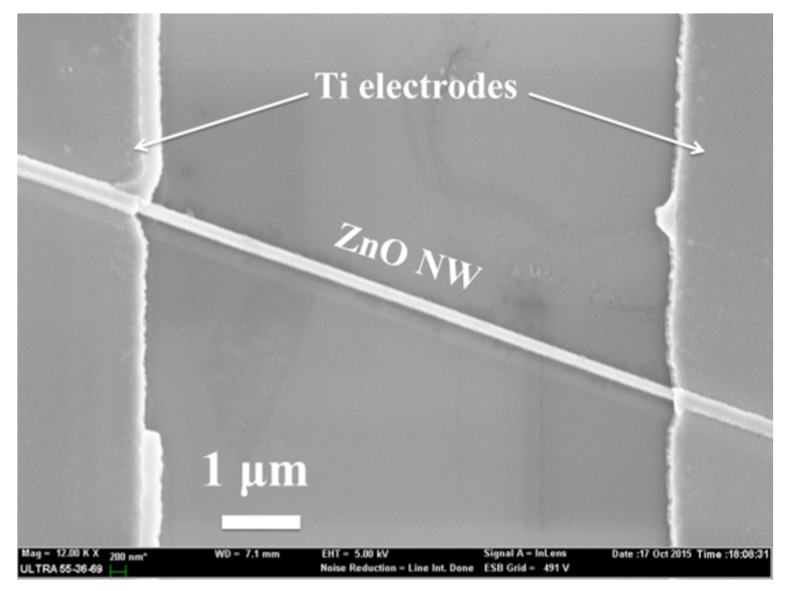
Top-view SEM images of the single ZnO NW–based resistive random access memory (RRAM) cell.

Thin gold films (~2 nm and ~ 4 nm) were firstly evaporated onto the Si(100) substrate prior to an 800 °C annealing for 10 minutes to form discrete gold nano-particles as catalysts. The ZnO NWs were then grown by a vapor-liquid-solid (VLS) method at 975 °C with a mixture of ZnO and carbon powder as precursor. Subsequently, the argon plasma treatment was applied to the as-grown nanowires with 100 W at 120 Pa for 240 s. The morphologies of the as-grown nanowires are shown in Figure 7. Compared with the ZnO nanowires grown with ~4 nm gold films, the ZnO nanowires grown with ~2 nm gold films have smaller diameter in a lower density that ensures the plasma treatment be employed even to the root of the nanowires. While the root of the ZnO nanowires with ~4 nm gold films should be protected from plasma treatment by the coverage of surrounding materials. The inhomogeneous plasma treatment effects should be obtained as what we observed in Figure 6a. To fabricate a single ZnO NW–based RRAM cell, horizontal ZnO NW memory was selected because its fabrication procedure would be less complex compared with that for a vertical ZnO NW memory. Additionally, the considerations of switching mechanism would be the same for the two structures. Therefore, the nanowires were released from the substrates by ultrasonic vibration prior to the dispersion onto the SiO_2_/Si(100) substrate. Two titanium electrodes spacing ~3 μm were subsequently sputtered and contacted with ZnO NW on its two terminals to form a memory cell as shown in Figure 9. To modulate the switching voltages and the resistance of the binary storage states, changing the plasma-treatment parameters or shortening the distance between the two electrodes might be tried in the consequent experiments.

The *I*–*V* characteristics and the other switching properties of the RRAM cells were characterized using a semiconductor characterization system (4200-SCS; Keithley, OH, USA). To evaluate the effects of plasma treatment, we measured the untreated memories and the treated memories in the same environment to ensure the protons and the absorbed species have the same effects on the memory cells. The morphology of the ZnO NWs and the single ZnO NW–based memory cell was observed by a scanning electron microscopy (Ultra-55; Zeiss, Oberkochen, Germany). PL spectra with 325 nm as excitation wavelength were also investigated for a better understanding of the defects.

## 4. Conclusions

The effects of plasma treatment on the resistive switching of a single ZnO NW have been investigated. The plasma treatment enhances not only the homogeneity but also the reproducibility of the resistive switching by the plasma-produced oxygen vacancies to average out the stochastic movement of individual oxygen vacancies. The plasma treatment could also reduce switching voltages due to the increased oxygen vacancies facilitating resistive switching. Additionally, inhomogeneous plasma treatment on the two terminals may result in self-rectification in the memory cell by weakening the rectification of the Schottky barrier at one terminal.
